# Mapping clinical governance to practitioner roles and responsibilities

**DOI:** 10.1108/JHOM-02-2020-0065

**Published:** 2020-12-18

**Authors:** Maureen Alice Flynn, Niamh M. Brennan

**Affiliations:** Health Service Executive , Dublin, Ireland; University College Dublin , Dublin, Ireland

**Keywords:** Clinical governance, Practice theory, Practice, Management, Governance, Practitioners, Praxis

## Abstract

**Purpose:**

While clinical governance is assumed to be part of organisational structures and policies, implementation of clinical governance in practice (the praxis) can be markedly different. This paper draws on insights from hospital clinicians, managers and governors on how they interpret the term “clinical governance”. The influence of best-practice and roles and responsibilities on their interpretations is considered.

**Design/methodology/approach:**

The research is based on 40 in-depth, semi-structured interviews with hospital clinicians, managers and governors from two large academic hospitals in Ireland. The analytical lens for the research is practice theory. Interview transcripts are analysed for practitioners' spoken keywords/terms to explore how practitioners interpret the term “clinical governance”. The practice of clinical governance is mapped to front line, management and governance roles and responsibilities.

**Findings:**

The research finds that interpretation of clinical governance in praxis is quite different from best-practice definitions. Practitioner roles and responsibilities held influence practitioners' interpretation.

**Originality/value:**

The research examines interpretations of clinical governance in praxis by clinicians, managers and governors and highlights the adverse consequence of the absence of clear mapping of roles and responsibilities to clinical, management and governance practice.

## Introduction

The term “clinical governance” was first used in 1998 ( Department of Health, 1998 ; Scally and Donaldson, 1998 ) to promote a system for delivery of safe quality care. Since then, practitioners, managers, researchers and policymakers have used the term “clinical governance” in ways that reflect different meanings and interpretations. Cleary and Duke (2019) highlight the continuing need for a clinical-governance agenda using a case of clinical-governance breakdown; while Gopal and Ahmad Kamar (2020) focus on the importance of clinical governance during the COVID-19 pandemic. Arising from their bottom-up study of clinical governance with healthcare professionals at one Dutch hospital, Veenstra *et al.* (2017 , p. 1) recommend further research “involving the perspectives of managers and policy makers”. This study answers their call by exploring in 40 interviews with clinicians, managers and governors their understanding of clinical governance at two large Irish academic hospitals.

Practice theory is useful in envisaging how practitioners take and shape (best) practices to create their clinical governance roles and responsibilities in praxis. Practice theory and its core tenets, practices, practitioners and praxis, provide a suitable analytical lens, particularly for studies using interpretive, interview-based methods.

The research contributes to the prior literature by extending:

1. Brennan and Flynn's (2013) analysis of 29 best-practice definitions of clinical governance, to examine interpretations of clinical governance in praxis.
2. Veenstra *et al.* (2017) by including managers and governors, as well as clinicians, in the sample of participants.

The findings highlight confusion and uncertainty for practitioners seeking to apply clinical governance (best) practices in their daily hospital activities. Using a single umbrella (catch-all) term compounds the problem and does not assist in explaining (best) practice. Findings confirm the need for clearer distinction in praxis between clinical practice, clinical management and clinical governance roles and responsibilities originally proposed in Brennan and Flynn (2013) . Findings reveal how the lack of clarity between the three categories of roles and responsibilities inhibits consistent interpretation of and implementation of clinical governance, clinical management and clinical practice in praxis.

Given the variation in interpretation by clinicians, managers and governors, the study points to an opportunity to strengthen interpretation of the term by clarifying clinical governance by reference to roles and responsibilities. Such a clarification would help each practitioner role-and-responsibility category to understand how practitioners contribute to clinical governance arrangements for the hospital.

## Literature review

This section examines prior research on clinical governance, the theoretical lens – practice theory – and what might influence practitioners' interpretations of clinical governance.

### What is clinical governance?

The United Kingdom (UK) Department of Health (1998) and Scally and Donaldson (1998 , p. 61) define clinical governance as “a system through which NHS organisations are accountable for continuously improving the quality of their services and safeguarding high standards of care by creating an environment in which excellence in clinical care will flourish”. Clinical governance is not a unitary concept, with variation in application and many constituent parts (e.g. Baker, 2003 ; Gauld, 2014 ; Som, 2009 ; Walshe, 1998 ). Prior research indicates that the concept, as well as the practice, of clinical governance has undergone significant development, leading to complexity and confusion.

The original clinical governance definition does not distinguish between how the system might apply across three categories of practitioner roles and responsibilities: Clinicians, managers and governors ( Baker, 2003 ; Degeling *et al.* , 2004 ; Som, 2004 , 2009 ; Veenstra *et al.* , 2017 and Walshe, 1998 ). To address this confusion, Brennan and Flynn (2013) apply content analysis to 29 definitions of clinical governance from the perspective of those charged with practice, management and governance, reflecting the importance of clear division between practice, management and governance roles, leading to more effective implementation of clinical governance in praxis.

### Practice theory

**Figure 1:**
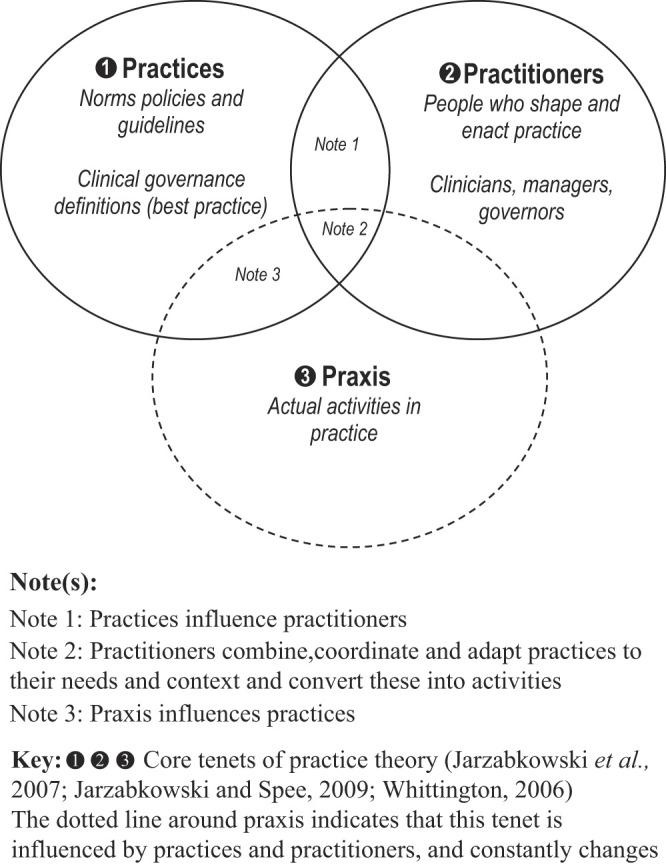
Core tenets of practice theory

Practice theory seeks to explain the relationship(s) between human action, on the one hand, and “the system” on the other ( Ortner, 2006 ). Whittington (2006) identifies the core tenets of practice theory: “practices”, “practitioners” and “praxis”. Figure 1 illustrates the relationships between these three core tenets, (best) practices, practitioners and praxis (activities in practice). Practitioners draw upon (best) practices to act which, in turn, generates praxis ( Jarzabkowski *et al.* , 2007 , p. 10). (Best) practices influence practitioners who combine, coordinate and adapt practices to their needs and context and convert these into praxes. In turn, through a feedback-loop adaptation process, praxis can shape (best) practices.

Practice theory is suitable for analysing the practice of clinical governance in hospitals due to the complexity of the relationships and the number of practitioners involved ( Feldman and Worline, 2016 ). We use the term “practitioner” throughout, to refer to the collective of clinicians, managers and governors, i.e. people engaged in clinical practice, clinical management and clinical governance. We also use the term “practitioner” to refer to interviewees, the participants in this research.

### Factors influencing practitioner interpretations

The original Department of Health (1998) / Scally and Donaldson (1998) definition of clinical governance is silent on “how”, “by whom” and at “what levels” clinical governance is to be achieved. Factors influencing clinical governance in praxis include best practice, practitioner role-and-responsibility category and practitioner hospital.

Practices refer to the shared understandings, rules, languages and procedures that guide and enable human activities. These shared understandings can be referred to as “best practice”. The Irish health services operate through a national body, the Health Service Executive (HSE). National policy and guidance documents assist service providers in applying clinical governance frameworks (Health Information and Quality Authority (HIQA), 2012 ; HSE, 2014 ; Flynn *et al.* , 2015 ; Gauld *et al.* , 2017 ). Unlike the UK, these do not have legislative backing ( Committee on the Future of Healthcare, 2017 ).

By reference to roles and responsibilities, Brennan and Flynn (2013) differentiate between three categories of practitioner roles and responsibilities: front-line practitioners, managers and governors. Practitioner influence has been found to be important in the interpretation of clinical governance. Davies *et al.* (2000) identify the different professional cultures (backgrounds / experience) of health service managers and medical professionals and point to the importance of a degree of “cultural fit” between these two key groups in clinical governance cultural transformation. Clinical governance is central to clinical directors' [1] work ( Gafoor *et al.* , 2020 ). Som (2009 , pp. 108-109) finds that “depending on their organisational level, the directorate in which they are working, their professional background and the responsibilities they have handled and their current job, staff vary in their interpretation of clinical governance”. Freeman and Walsh (2004 , p. 335) find directorate-level manager perceptions of achievement in 54 clinical governance items to be significantly lower than those of their board-level colleagues on all domains other than improving clinical governance performance. Jones *et al.* (2017) highlight the importance of board members in clinical governance.

Type of hospital has also been found to influence clinical governance ( Hogan *et al.* , 2007 ; Pronovost *et al.* , 2018 ). Karassanvidou *et al.* (2011 , p. 236) find type of hospital relevant in terms of its organisational culture. While not directly examining hospital influence, Brault *et al.* (2015) identify the hospital accountability structure as a clinical-governance lever.

## Research questions and research methodology

Extending the work of Brennan and Flynn (2013) , this research examines what the term “clinical governance” means to practitioners in their everyday activities, i.e. their interpretation of clinical governance in praxis by comparison with clinical governance best practice.

The research questions are:

RQ1.
How does the interpretation of clinical governance in praxis compare with best-practice definitions?

By reference to:

RQ1a.
Keywords/terms used in definitions but not used by practitioners?

RQ1b.
Keywords/terms in common between best-practice definitions and those used by practitioners?

RQ1c.
Keywords/terms used by practitioners in praxis but not in definitions?

RQ1d.
The frequency of usage of keywords/terms in definitions compared with by practitioners in praxis?

RQ2. How does the interpretation of clinical governance in praxis map to practitioner roles and responsibilities?

### Interviewees

The research conducts 40 in-depth semi-structured interviews, informed by the research questions and Brennan and Flynn's (2013) content analysis of 29 definitions of clinical governance. Interviews focus on practitioners' understanding of the term “clinical governance”. Pilot interviews ( *n* = 4) validate the data collection methods. Pilot study data is not included in these research findings.

Practitioners ( *n* = 40) from two sites, Hospital A ( *n* = 20) and Hospital B ( *n* = 20), participate in the study. The hospitals are large university tertiary referral hospitals, providing acute medicine, surgery and national speciality services. The research selects the two hospitals on the basis that the hospital (1) has a hospital board, (2) is an acute general hospital and (3) is a large academic university hospital. Seven of the 48 publicly funded hospitals in Ireland meet the three inclusion criteria. The three categories of practitioner roles and responsibilities comprise: (1) front-line clinicians, (2) managers and (3) governors (see Table 1 ). Clinicians include medical doctors, advanced nurse practitioners, pharmacists and therapy professionals. Through a nominated link person, the interviewer (first-named author) invites hospital managers with single roles (e.g. CEO, Director of Nursing, Director of Human Resources, Director of Finance) to participate. The link person purposively connects with clinician participants and non-executive board members for their experience using a snowball approach ( Richards and Morse, 2013 ).

### Interviews

The interviewer asks practitioners two open-ended interview-guide questions: (1) what do you understand by the term “clinical governance”? and (2) to what extent has clinical governance featured in your roles?. The interviewer audio records and transcribes the interviews, along with interviewer notes drafted during and after the interviews. The interviewer returns transcripts to participants for checking. The interviewer uploads interview transcripts in NVivo 10 for coding and reviews interview transcript responses line-by-line.

### Operationalising the constructs

The research includes two constructs: (1) clinical governance best practice and (2) interpretation of clinical governance in praxis. For RQ1 , the research applies a coding architecture using *a priori* keywords/terms from Brennan and Flynn's (2013) 29 clinical governance definitions. The research catalogues the 1,000 most frequent keywords used by practitioners to describe clinical governance. To operationalise best practice, the study identifies keywords relating to roles and responsibilities in Brennan and Flynn's (2013) 29 definitions of clinical governance. The research operationalises praxis through the keywords/terms spoken by practitioners to describe their clinical governance roles and responsibilities. The research analyses interview transcripts using keyword and keyterm content analysis ( Krippendorff, 2013 ). The unit of analysis is “words” (single word) and “terms” (combination of words) interviewees use to describe their clinical governance roles and responsibilities. During the analysis, minor variations in *a priori* keywords/terms and new emerging keywords/terms are added to the architecture. Understandably, interviewees use the words “clinical” (115) and “governance” (102) most commonly. As these two words are the kernel of the concept being described, they are not included in the content analysis counts.

### Analysis of the data

This research uses rank orders of the frequency of usage (i.e. number of mentions) by practitioners of keywords/terms as proxy indicators of the prominence for practitioners of the various clinical governance roles or responsibilities (as direct observation was not feasible). Rank ordering of ordinal data is more robust to outliers (by ignoring step changes created by frequencies) ( Kerlinger and Lee, 2000 ).

## Discussion of findings

This section provides the study findings by reference to the two research questions.

### Comparison of best-practice keywords/terms and usage in praxis (RQ1)

The number of keywords/terms used by practitioners to describe clinical governance evidences the impact of including a wide number of activities and functions within one concept. Table 2 shows that practitioners use 71 keywords/terms in their interpretations of clinical governance in praxis.

Of the 40 keywords in definitions, six are not used by practitioners (see Table 2 ) ( RQ1a ). These keywords are not commonly used in the definitions. We believe this is because practitioners do not favour command-and-control (“regulated professional”, “compliance”, “obligation”) and business-orientated terms (“inputs”). However, we acknowledge this is a broad interpretation based on our assumption which others may not share. Mintzberg (2017 , p. 94) concurs with our view when he observes: “The field of health care may be appropriately supplied by businesses, but in the delivery of its most basic services, it is not a business at all, nor should it be run like one. At its best, it is a calling”. It is somewhat surprising that “customer participation” or the more common term “patient involvement” is not used by the practitioners. Practitioners use 34 keywords/terms in common with the 40 (85%) keywords/terms in Brennan and Flynn's (2013) 29 clinical governance definitions (best practice) (see Table 2 ) ( RQ1b ). Practitioners add extensively to keywords in definitions with 37 additional keywords/terms of their own (see Table 2 ) ( RQ1c ).

**Figure 2:**
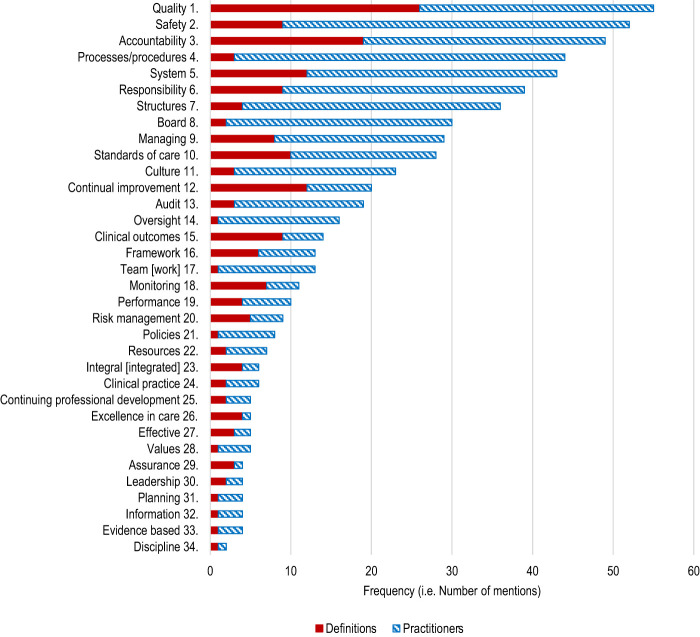
Rank order of clinical governance keywords/terms in common between definitions (best-practice) and used by practitioners in praxis ( RQ1b , RQ1d )

Addressing RQ1b and RQ1d , Figure 2 rank orders the frequency of practitioner usage of the 34 keywords/terms and compares it with the rank ordering of usage of the 34 keywords/terms in Brennan and Flynn's 29 clinical governance definitions. The keywords “1. quality”, “3. accountability”, “5. system” and “12. continual improvement” feature most prominently in the definitions, while “2. safety”, “4. processes/procedures”, “7. structures” and “5. system” are most prominent in practitioners' descriptions of clinical governance.

**Figure 3:**
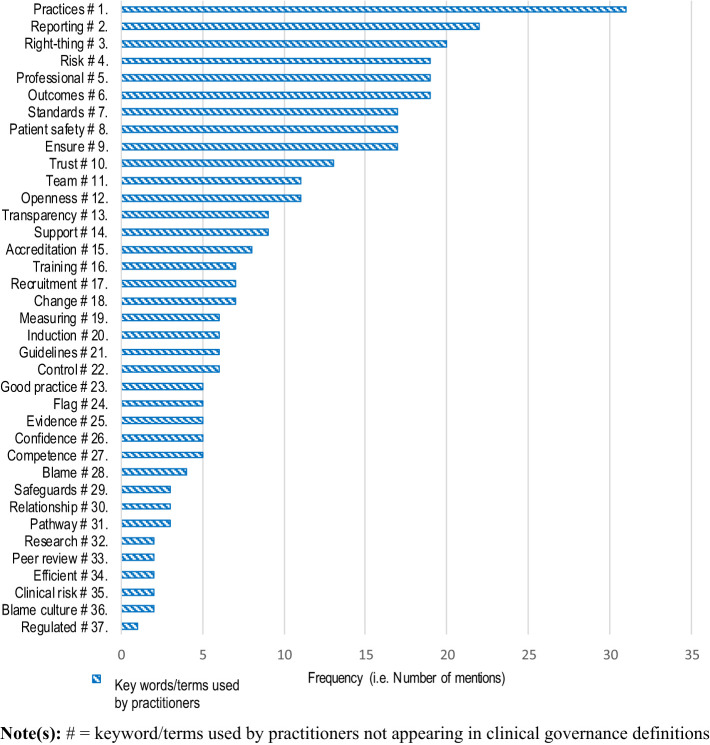
Rank order of new keywords/terms used by practitioners in their interpretation of clinical governance (praxis) not in best-practice definitions ( RQ1c )

Figure 3 rank orders the frequency of practitioner usage of the 37 keywords/terms added by practitioners. Many of these keywords/terms reflect the practical outputs of the application of clinical governance, such as “2. reporting”, “6. outcomes”, “8. patient safety”, “10. trust”, “12. openness”, “13. transparency” and “18. change” – again, potentially signifying practitioners' wish to move away from command-and-control language. However, practitioners also add more negative keywords/terms such as “9. ensure”, “22. control”, “28. blame”, and “36. Blame culture”, evidence of an “on top” hierarchical, command-and-control environment ( Mintzberg, 2012 , p. 6). There is a strong focus in the new keywords on people-related aspects of clinical governance such as “14. support”, “16. training”, “17. recruitment”, “20. induction”, “26. confidence” and “27. competence”.

**Figure 4:**
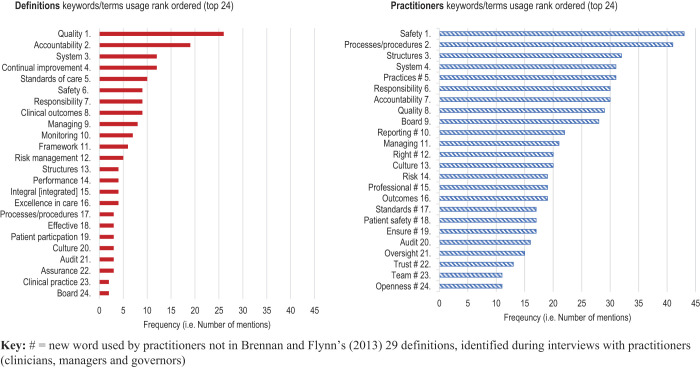
Comparison of rank ordering of most common usage of keyword/terms in definitions (best-practice) and used by practitioners (praxis) ( RQ1d )

Addressing RQ1d , Figure 4 compares the 24 most-frequent keywords/terms in the definitions with the 24 most-frequent keywords/terms used by practitioners. These 24 keywords/terms represent the top 50% of keywords most frequently used by practitioners. If clinical governance definitions influence practitioner interpretation, the rank order of definitions/practitioner terms in both columns in Figure 4 should be similar. However, Figure 4 shows that the frequency of clinical governance keywords/terms used by practitioners does not appear to be influenced by best-practice clinical governance definitions. It may be that the norms and expectations observed and experienced during practitioners' careers have a stronger influence, perhaps because the definitions are found to be somewhat vague and limited when considering practical application of practitioner roles and responsibilities. This suggests that practitioners' interpretation of clinical governance may be obtained more from the ground rather than from official policy and definitions. Practitioners “pick-up” the idea of clinical governance by observing peers, from experience, and from their own, what can be called, on-the-ground practice. As Mintzberg (2013 , p. 174) suggests, “if we really want to understand what has happened to management, then we would do well to get down on the ground […]”.

### Influences of practitioner roles and responsibilities on interpretation of clinical governance in praxis (RQ2)

RQ2 examines influences of practitioner roles and responsibilities on the interpretation of clinical governance in praxis. Keywords/terms spoken by practitioners are analysed by reference to the three categories of practitioner roles and responsibilities (see Tables 2 and 3 ). Table 2 shows that the 71 keywords/terms are used, with managers using fewer keywords/terms. Table 3 analyses the frequency of usage (748 usages in total) across the three categories of practitioner roles and responsibilities and by reference to individual keywords/terms. Frequency of usage by practitioners varies from 43 times (“safety”) to 1 time (six keywords/terms). The rank ordering in Table 3 shows marked variation between the frequency of usage of keywords/terms.

The 71 keywords/terms used by interviewees are allocated between front-line delivery, management and governance roles and responsibilities. The purpose is to map clinical governance to the three categories of roles and responsibilities (see Table 3 ). As previously acknowledged, the classification of keywords/terms into three role-and-responsibility categories involves judgement; some terms (e.g. “risk management”, “leadership”, “blame”, “information”) may fit under more than one heading. The subjective nature of the analysis is such that other researchers might produce different analyses.

### Summary of findings

The insights suggest that, while there is some awareness of clinical governance, there is still much confusion and uncertainty about who is responsible and how to activate the system at various hospital role-and-responsibility levels.

In total, 77 unique keywords/terms are used across the definitions and descriptions of clinical governance in praxis. The research finds that practitioners use many more keywords/terms in their own descriptions of clinical governance (71) than provided in definitions (40). Practitioners include 37 additional keyword/terms not in Brennan and Flynn's (2013) 29 definitions of clinical governance. Of note, most keywords/terms (34–85%) in best-practice definitions are included by practitioners in their personal description of what clinical governance means to them, even though several indicate that they are not aware of or do not refer to definitions. There are a small number (6–15%) of keywords/terms in definitions (best practices) describing clinical governance not used by practitioners, such as “registered professional”, “compliance”, “obligation”, “inputs”, “customer participation” and “clinical judgement”. Practitioners are possibly more conscious of best-practice governance arrangements evident in the commonality of use of clinical governance keywords/terms between practitioners' and Brennan and Flynn's (2013) clinical governance definition, for example, “structures” and “system” of “accountability”, “responsibility”, “culture”, “audit” and “oversight”. Of note, the lowest number (6 – Table 2 ) of new keywords/terms pertains to the governance role category. A larger number of new keywords/terms pertain to front-line-delivery roles and management (15/14 respectively – Table 2 ). The study reveals that the umbrella term has not been mapped to people and roles and responsibilities within Hospital A and Hospital B.

Almost all participants (36–90%) talk about the extent that clinical governance features in their role, be they clinician, manager or governor. Practitioners describe clinical governance activities as “absolutely” a feature of their role, “central”, “huge”, “permeates”, “a lot”, “part and parcel”, “a feature of day-to-day professional life”, incorporated in “every part of my role”, “it's ever present, it's every single day, many nights and some weekends” and “it's a massive part of my working life now here”. All clinical directors mentioned the constraints of time impacting on the extent they can focus on clinical governance due to their clinical work commitments. Thus, implicitly, they see clinical work and clinical governance as separate activities. This is sub-optimal. Clinical practice and clinical governance should be hand-in-glove connected, for clinical governance to operate effectively.

In praxis, practitioners are only partially influenced by definitions of clinical governance because of the “picked-up” nature of their construction of clinical governance. Practitioners are aware of, but confused by, clinical governance. They emphasise trust and the calibre of people more than accountability and independent review. However, despite this, clinical governance is ever present in their work and thinking.

## Conclusion

The findings affirm variation in interpretation, understanding and confusion around the use of the term clinical governance. The findings signal the disparity and confusion among practitioners, particularly around front-line clinical practice and management roles and responsibilities. Findings support the differentiation between front-line delivery, management and governance roles and responsibility and support using Brennan and Flynn's (2013) three separate definitions.

### Limitations

Gaining insights from hospital practitioners' experiences answers the call for exploratory research reflecting the context of clinical governance ( Veenstra *et al.* , 2017 ). The findings are limited to experiences of practitioners in the healthcare services where they have worked. Some interviewees have overlapping roles, whereas the study categorises them into one of three role categories. For example, some clinicians are also clinical directors ( *n* = 10), while some clinicians ( *n* = 4) have roles as governors (members of the board of directors). Because many interviewees do not fall uniquely into the three role categories (clinician, manager or governor), it is not possible to map the words they use into the three role categories. This reflects the complexity of healthcare with multiple overlapping roles and responsibilities. In addition, clinical directors experience the predicament of their management role affecting relationships with colleagues. All suggest that they need more time to focus on clinical governance (perceived as management functions) but, for clinical credibility among colleagues and managers, they also indicate (unlike other countries where clinical directors are full-time) that the best arrangement would be to retain some clinical practice, but for something less than the current 50% of their time. Practitioners identify systems of accreditation (voluntary or required by regulators) as one positive initiative assisting clinical directors in bringing attention to clinical governance and its execution. Despite some confusion, ambivalence and juggling a myriad of roles, this study reveals that clinical governance is ever present in practitioners' work and thinking. In this environment, there is a risk of practitioners suffering from role confusion and conflict. The scope of the study does not provide for the inclusion of the perspectives of patients or the public on the praxis of clinical governance. However, careful attention is given to identifying clinicians’, managers’ and governors’ thoughts on patient perceptions and experiences during the analysis.

### Implications for theory and practice

The confusion around the definition of clinical governance and its application in practice is evident in the absence of a standardised approach to interpretation by practitioners participating in this research. The findings indicate that some practitioners are hesitant and, on occasion, find it difficult to articulate their role in clinical governance. Despite this, their personal descriptions reflect several features of clinical governance set out in (best) practice definitions. Achieving effective clinical governance requires a collaborative effort between clinicians, managers and governors being clear about their separate and distinct roles. Use of “catch-all” understanding of clinical governance, regardless of roles/responsibilities or by whom within the hospital it is to be applied, compounds the confusion. For effective clinical governance, it is important that there be division of duties between front-line delivery roles, management roles and governance roles. It is a fundamental principle of governance that governors cannot oversee and monitor their own work. The praxis of clinical governance described by practitioners in this study does not distinguish between front-line delivery, management and governance functions. To embed clinical governance fully in hospitals, it is necessary to clearly articulate the roles and responsibilities relating to clinical governance. Providing three separate definitions provides clarity on the operation of clinical governance in each role (as set out in Brennan and Flynn, 2013 ). These distinctions will help to clarify roles and responsibilities in the accomplishment of clinical governance and concomitant increase in patient safety.

The difference in understanding this study reveals is an important consideration for executive management teams and boards. Heretofore, most attention has been given to independence in thinking and challenge by having both internal and external non-executive membership of hospital boards of directors. This study suggests that it is also important to consider further diversity by having a combination of those who have the benefit of clinical experience during their career and those with no clinical experience among both the executive and non-executive board member groups.

### Concluding comment

There is an absence of mapping of clinical governance to the roles and responsibilities of the parties expected to execute clinical governance. The absence of mapping, and therefore discernibility of clinical governance roles and responsibilities, leads to confusion for those expected to execute them. The use of the term clinical governance is losing prominence. Gauld and Horsburg (2020) provide evidence that the clinical governance agenda has stalled. Alternatives are being proposed for example “governance for quality” and “governance of quality and safety” ( Flynn *et al.* , 2015 ). Variations of the term are equally problematic.

The study findings show that best-practice definitions of clinical governance are not alone in influencing practitioners in their interpretation of clinical governance. It is apparent that providing a “catch-all” definition is not enough, particularly when available definitions are considered confusing. It is evident that the calibre of staff providing role models, and a culture supportive of clinical governance, are equally a way of developing the norms and expectations that construct practice. In the praxis of clinical governance, practitioners do not adequately distinguish between practice, management and governance functions, particularly when they are “juggling roles”. Obtaining enough independent review to seek assurance and inform board oversight is a priority. However, how accountability (a core element of clinical governance) is executed has the potential to affect the creation of cultures of reporting and openness or not and, ultimately, the “north star” of quality and safety for patient care in hospitals. There is an increasing awareness in financial services of the importance of precisely mapping people's roles and responsibilities. This is aided by arrangements such as the UK's senior managers' regime or the forthcoming Irish Senior Executive Accountability Regime ( KPMG, 2018 ). The regime provides a tool that helps senior leaders to be crystal clear about their responsibilities and can therefore better manage their duties. Mapping clinical governance to clinician, manager and governor roles and responsibilities has the potential to enable responsive cultures of person-centred healthcare.

## Figures and Tables

**Table 1:** Overview of interviewees

	Hospital A	Hospital B	Total
Clinician	8	9	17
Manager	3	5	8
Governor	9	6	15
Total	20	20	40

**Table 2:**
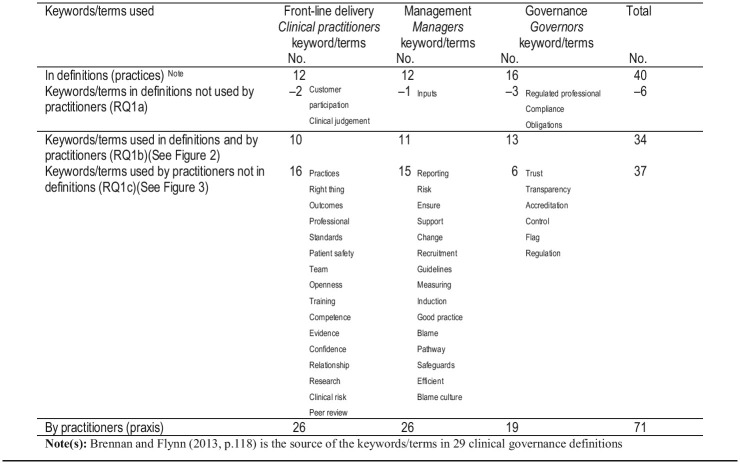
Keywords/terms in 29 definitions of clinical governance (best-practice) and practitioner descriptions of clinical governance (praxis)

**Table 3:**
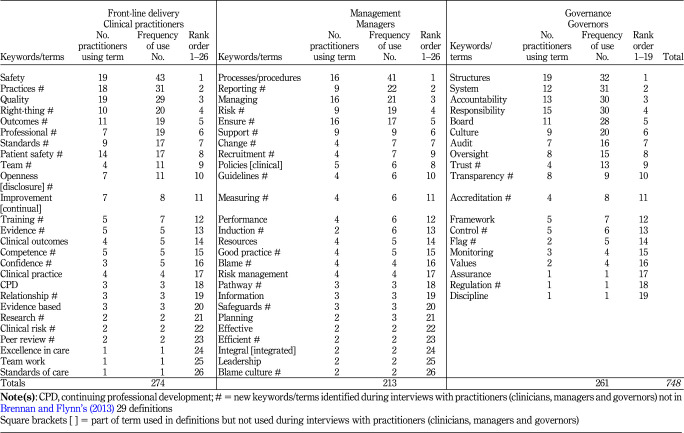
Frequency/ranking of keywords/terms used by practitioners to describe the practice of clinical governance, grouped by front-line delivery, management and governance ( RQ2)

## Note

1. A medical doctor who has a leadership role for one or more specialties within a hospital. The primary role is to manage and plan how services are delivered and contribute to the process of strategic planning, influencing and responding to organisational priorities.
